# Dynamic benefits of finding the silver lining: secondary control as a buffer against declines in well-being during COVID-19

**DOI:** 10.1007/s11031-026-10195-9

**Published:** 2026-02-06

**Authors:** Matthew Pierce, Katherine Duggan, Clayton Hilmert, Heather Fuller, Jeremy Hamm

**Affiliations:** 1 Department of Psychology, North Dakota State University, Fargo, USA; 2 Department of Human Development and Family Science, North Dakota State University, Fargo, USA

**Keywords:** Secondary control, Well-being, Financial hardship, Dynamic shifts

## Abstract

Although secondary control (SC) beliefs reflect a motivational resource that can protect well-being, little is known about the potential benefits of dynamic shifts in SC in response to socioeconomic hardships. The present study analyzed data from an online national COVID study that collected 5 waves of data from a representative sample of U.S. adults aged 18–80 during the first two years of the pandemic (*n* = 292). Multilevel models assessed how within-person changes in SC were associated with shifts in mental health (perceived stress, depressive symptoms) and well-being (affect, life satisfaction, meaning in life, personal growth) and the extent to which this relationship depended on increased financial hardship. Results indicated that increases in SC were associated with adaptive shifts across all included outcomes. Financial hardship moderated the link between SC, perceived stress, and personal growth, such that SC was beneficial during periods of financial hardship. Findings provide evidence for the role of dynamic changes in SC in protecting well-being during intractable life circumstances.

**Supplementary Information:**

The online version contains supplementary material available at 10.1007/s11031-026-10195-9.

## Introduction

The COVID-19 pandemic set the stage for a multitude of rapid life-altering and stress-inducing personal and societal hardships, including isolation, job loss, fear of personal infection, and concerns over economic downturn (Fisher et al., 2020; Shanahan et al., 2020). The constraints on personal control imposed by the pandemic undermined people’s mental health and psychological well-being, as people are motivated to exert control over their environments whenever they can because such control promotes safety, health, and comfort (Morling & Evered, 2006). When control over one’s external environment is constrained in such a manner, theories of motivation and control suggest that people may turn inwards towards internal processes that maintain a sense of control (Rothbaum, 1982; Lachman, 2006; Heckhausen et al., 2019).

These internal processes are referred to as *secondary control* (SC); this is in contrast to *primary control* (PC) processes that involve influence over external life circumstances (Rothbaum et al., 1982; Morling & Evered, 2006). SC reflects an acceptance and/or reframing of the various challenges that an individual might face in order to maintain a sense of control, encapsulated well in phrases such as “every cloud has a silver lining” or the line in Niebuhr’s Serenity prayer that addresses “[accepting] the things I cannot change” (Chipperfield et al., 2012; Rothbaum, 1982; Morling & Evered, 2006). People are motivated to seek out SC as a buffer against helplessness when confronted with situations that might otherwise trigger this response (Rothbaum, 1982; Morling & Evered, 2006). There is evidence that, when faced with uncontrollable circumstances, individuals with higher levels of perceived SC also tend to have higher well-being when compared to individuals with lower levels of SC (Chipperfield et al., 2012).

While research shows that such individual (between-person) differences in levels of SC are linked to better well-being, less is known about how within-person fluctuations in an individual’s SC affect changes in well-being. This is a crucial gap in the literature because, as indicated by events such as the COVID-19 pandemic, perceptions of control are a fluid process that can and do shift over time, across life circumstances, and in response to environmental factors (Hamm et al., 2025; Hamm Shane et al., 2023b; Robinson & Lachman, 2020). The extent to which socioeconomic-related hardships may impact the relationship between SC and well-being also remains unknown. Shifts in socioeconomic status (SES) that reflect gains or losses in external control may reflect a key moderator in determining the circumstances under which people benefit most from increases in SC. Thus, the present study used 2-year longitudinal data from a national U.S. sample to test (a) whether within-person increases in SC were linked to corresponding shifts in mental health (perceived stress, depressive symptoms), hedonic well-being (positive and negative affect, life satisfaction), and eudaimonic well-being (purpose in life, personal growth) during the pandemic and (b) the extent to which this relationship depended on changing socioeconomic circumstances involving shifts in financial hardship.

## Secondary control and well-being

Prior research on SC has supported the theoretical proposition that SC can facilitate well-being. Individual (between-person) differences in SC have been shown to predict better psychological well-being, including less perceived stress, fewer depressive symptoms, more positive affect, and less negative affect (Chipperfield et al., 2017). For example, a study by Swift and Chipperfield (2013) found that individuals with higher SC reported higher levels of life satisfaction and positive emotion. Subsequent research has also documented positive associations between SC and well-being (Helzer & Jayaqickreme, 2015; Parker et al., 2022). Helzer and Jayawickreme (2015) found that SC was particularly beneficial for maintaining day-to-day subjective well-being; individuals who endorsed SC-related strategies also tended to report greater subjective well-being. Parker et al. (2022) found that SC beliefs, including those related to adjustment and acceptance, predicted increased positive emotion and reduced perceptions of stress in a sample of young adults facing setbacks during the transition to college. Prior studies had found similar results in the context of college achievement settings, such that secondary control tended to be beneficial for students’ well-being (Credé & Niehorster, 2011; Prihadi et al., 2018) These findings collectively point to SC as a self-regulatory motivational resource that supports multiple indicators of well-being, including mental health (perceived stress and depressive symptoms) and hedonic well-being (affect and life satisfaction).

This body of evidence provides support for the relationship between individual (between-person) differences in SC and well-being. However, little remains known about the extent to which this relationship is dynamic in nature due to a dearth of studies examining SC and well-being at the within-person level. That is, prior research has largely yet to examine the role of changes or fluctuations in SC over time. Most research on SC to date has evaluated SC at a single timepoint, neglecting the ecological reality that SC can, and presumably does, change over time. The few studies that have been conducted in this area are promising, however. For instance, a 2021 micro-longitudinal study by Wang and colleagues examined stressors in U.S. college students during the COVID-19 pandemic via daily diary studies and found that within-person increases in SC predicted both same-day and next-day increases in positive affect and decreases in negative affect. Another examined the health-related quality of life among childhood cancer survivors over a five-year period (Fisher et al., 2021). The study found that participants who experienced positive shifts in SC coping experienced increases in health-related quality of life. However, while prior work provides preliminary evidence for the potential role of changes in SC over time, further research is needed to systematically evaluate the extent to which SC varies over time in more representative adult lifespan samples and whether these fluctuations in SC have implications for a broader suite of well-being outcomes within the context of low-control environments such as the pandemic.

## Secondary control in low-control circumstances

Theories of motivation and control posit that people seek out means of SC when there are limited avenues for PC and one must instead internally adjust to external challenges (Morling & Evered, 2006; Chipperfield et al., 2017). The increased salience of SC in low control circumstances was explored in a series of studies by Thompson and colleagues that examined SC as a backup for PC; when PC was low, SC was more adaptive (Thompson et al., 1994, 1998). Their research with HIV-positive men found that higher levels of SC served a protective role in buffering against declines in mental health, notably depression (Thompson et al., 1994). A similar pattern emerged in the context of age-related changes in physical appearance (Thompson et al., 1998).

The role of SC in response to loss of external (primary) control has also been extensively examined in the context of aging, as proposed by Heckhausen et al.’s motivational theory of lifespan development. This theory posits that SC is used by individuals to preserve PC as goals become unattainable through age-related developmental losses (Heckhausen et al., 2010; Heckhausen & Shulz, 1995; Schulz & Heckhausen, 1996). Research into SC in older adults has also shown improved mental and physical well-being, including increased overall life satisfaction (Chipperfield et al., 2012; Haynes et al., 2009). A similar pattern has been observed with gender differences, with prior research arguing that a patriarchal society force women into a type of low-control circumstance. Previous research has shown that older women, when compared to men, tend to rely more than men on SC, and use more varied SC strategies when encountering health difficulties (Chipperfield & Perry, 2006; Chipperfield et al., 2007; Swift et al., 2008). A 2022 study by Toyama similarly found that older adults who endorsed multiple forms of SC exhibited greater levels of life satisfaction. Taken together, these findings provide evidence that individuals who tend to be afforded less external control over their lives may benefit most from increases in SC. This suggests that those experiencing low control circumstances in the form of socioeconomic losses may also disproportionately benefit from SC, but research has yet to systematically examine this issue.

Consistent with this idea, theoretical frameworks outside the control literature suggest that individuals experiencing lower-SES circumstances, such as those facing financial hardship, may benefit from SC, pointing to the potential moderating influence of financial hardship. Specifically, the Reserve Capacity Model pioneered by Gallo and Matthews (2003, 2011) suggests that people have a bank of psychosocial resources from which they can draw on to buffer against stressors. Their model suggests that the inherent stressors of a low-SES environment drain this bank of resources more than other environments. This suggests that people in low-SES environments are both more susceptible to daily stressors and are most benefited from additional psychosocial resources such as SC (Gallo & Matthews, 2003; Matthews & Gallo, 2011). Previous research provides some preliminary and indirect evidence that supports the contention that SC may have pronounced benefits in in low SES circumstances. For instance, research by Chen and Miller observed a buffering effect of a psychosocial composite that includes SC: shift-and-persist strategies. These shift-and-persist strategies emphasize adjusting oneself to better cope with stressors via reappraisal and acceptance (SC) while also persisting through adversity via optimism and a sense of meaning in life (Chen & Miller, 2012). This indicates the potentially beneficial impact of SC when experiencing SES-related hardships. Related research by Wadsworth and colleagues examined parent-child relationships and the influence of various control-related coping strategies used during times of financial strain. Their 2005 study found that greater levels of SC coping predicted lower hostility and depressive symptoms in the parent-child dyads, buffering the impact of economic strain and general life stress (Wadsworth et al., 2005).

Perceived financial hardship reflects an indicator of financial stress that may be particularly relevant in the context of SC. Measures of perceived financial hardship are posited to better reflect material conditions that may differ within and between individuals due to personal circumstances, lifestyle, geographic region, and health needs, and how one’s financial resources impact their perceived stressors in day-to-day life (Frankham et al., 2020; Whelan et al., 2001). Financial hardship is also a variable that may be more susceptible to dynamic changes over time. While income or education may shift more slowly, over the course of months or years with little change, financial hardship may fluctuate over short time periods. Financial hardship has also been linked to depressive symptoms and other indicators of mental health, and more strongly than other measures of SES (Butterworth et al., 2012; Lahelma et al., 2006). This suggests financial hardship may reflect an especially apt measure to consider for the role of SC during the COVID pandemic, a period of rapid change in financial circumstances. Though COVID-19 affected the entire population, its impacts were still felt more harshly during periods of economic loss and hardship (Nikolaidis, 2022; Vilar-Compte et al., 2022). This means that, if SC buffers against the negative impacts of a low-SES environment, increases in this self-regulatory resource should be especially beneficial during periods of hardship, whereas its absence should be especially detrimental.

Although previous research has examined more static differences in uncontrollable life circumstances, little remains known about how rapidly changing circumstances, especially fluctuations in financial hardship, play a role in moderating the relationship between SC and well-being. Open questions remain about how increases in SC in response to increased financial hardship may buffer against declines in mental health and well-being. This is especially the case for subjective measures of SES, such as financial hardship. However, research is needed to systematically test whether the within-person link between SC and WB depends on rapidly changes in financial hardship.

## Secondary control during the COVID pandemic

**SC and well-being during COVID** The COVID-19 pandemic reflects a unique set of circumstances to study the impact of SC on well-being during a period that precipitated significant fluctuations in external control and may have incentivized seeking out internal means of control. The pandemic created a situation in which, across populations, people’s external control was hampered due to pandemic-induced constraints (Fisher et al., 2020; Shanahan et al., 2020). Longitudinal studies that began at pandemic onset have shown that people tended to experience decreased primary (external) control, mental health and well-being during the early stages of COVID-19 (Fisher et al., 2020; Hamm et al., 2023b; Shanahan et al., 2020). This provides a grim “natural experiment” that created a set of low control circumstances during which the influence of SC on well-being may become more pronounced (Rutter, 2007). This makes it an ideal context to examine the relationship between dynamic, within-person changes in SC and well-being that are likely to occur in response to these challenging circumstances.

**SC**,** well-being**,** and financial hardship during COVID** Not all populations were equally impacted by the pandemic. A meta-analysis on studies conducted during the pandemic found that there was significant heterogeneity in how the pandemic influenced well-being across sociodemographic populations (Prati & Mancini, 2021). There is evidence that the well-being of individuals in low SES conditions was more adversely affected by COVID-19 (Nikolaidis et al., 2022; Prati & Mancini, 2021). Experiencing lower SES during the pandemic was linked to economic and resource insecurity and negative mental health consequences during the pandemic (Nikolaidis, 2022; Vilar-Compte et al., 2022). Increasing SC in response to these pandemic-related hardships may be especially beneficial in buffering against declines in well-being, but little is known about this moderated relationship. Longer-term data, collected over the course of years, would be required to fully encapsulate the changing nature of the relationship between SC and well-being as financial situations change in response to events such as the development of COVID-19 vaccines or various government responses. There also remain substantial gaps in the literature regarding SC during COVID-19. Though Wang and colleagues’, 2021 study took place during the pandemic, it only captures a brief time period at the beginning of the pandemic. Data on the dynamic link between SC and well-being through the course of the pandemic is limited and represents a unique avenue for examining SC.

## The present study

Using 2-year data from an online national COVID study, our first aim was to examine the extent to which within-person increases in SC predict corresponding shifts in central indicators of mental health (perceived stress, depressive symptoms), hedonic well-being (positive and negative affect, life satisfaction), and eudaimonic well-being (meaning in life, personal growth) during the COVID-19 pandemic (Chipperfield et al., 2017; Credé & Niehorster, 2011; Prihadi et al., 2018). Our second aim was to examine the moderating role of dynamic changes in financial hardships to determine whether the link between increases in SC and improved well-being was pronounced during low control periods that involved socioeconomic losses (increases in financial hardship). Consistent with previous literature, we hypothesized for Aim 1 that increases in SC would predict concurrent increases in mental health and well-being (e.g. Chipperfield et al., 2017; Parker et al., 2022). Consistent with control theory and the reserve capacity model, we hypothesized for Aim 2 that increases in SC will be a stronger concurrent predictor of well-being during circumstances that involve socioeconomic losses (vs. gains) in the form of increased financial hardships (e.g. Chen & Miller, 2013; Heckhausen et al., 2010).

## Methods

### Participants and procedures

The present study used 5-wave data from an online national COVID Study, an ongoing longitudinal study with a representative sample of 301 U.S. adults aged 18–80, mean 46. Data collection began during the early stages of the COVID-19 pandemic on April 16, 2020 (Wave 1). Two subsequent waves of data were collected early in the pandemic on May 1 (Wave 2) and June 17 (Wave 3). Subsequent follow-up data was collected 1 year (May 2021; Wave 4) and 2 years later (June 2022; Wave 5). Participants were recruited via Proflic.co and compensated for their time at each wave. Demographic, psychosocial, and health data was collected via online survey through the secure survey platform Qualtrics. Table 1 shows the correlations between each variable measured at the between-person level using person-mean data.

**Table 1:** Between-Person variable correlations

Variable	SC (pm)	Hardship (pm)	PS (pm)	DP (pm)	PA (pm)	NA (pm)	LS (pm)	MN (pm)	PG (pm)	Age (b)	Sex (b)	Edu (b)	Inc (b)
SC													
Hardship	−0.03												
PS	−0.40	0.40											
DP	−0.41	0.33	0.89										
PA	0.44	−0.28	−0.75	−0.74									
NA	−0.36	0.35	0.89	0.93	−0.69								
LS	0.42	−0.43	−0.68	−0.66	0.75	−0.64							
MN	0.54	−0.22	−0.61	−0.65	0.68	−0.56	0.70						
PG	0.46	−0.13	−0.38	−0.37	0.39	−0.32	0.36	0.57					
Age	0.10	−0.04	−0.29	−0.27	0.10	−0.27	0.07	0.02	−0.15				
Sex	−0.03	0.08	0.21	0.18	−0.2	0.21	−0.10	−0.07	0.07	0.06			
Edu	−0.02	−0.22	−0.17	−0.14	0.14	−0.15	0.25	0.09	0.07	0.18	0.04		
Inc	−0.02	−0.42	−0.14	−0.1	0.10	−0.10	0.26	0.13	0.13	−0.09	−0.02	0.16	

*SC *secondary control, *PS *perceived stress, *DP *depressive symptoms, *PA *positive affect, *NA *negative affect, *LS *life satisfaction, *MN *meaning in life, *PG *personal growth, (b) = measured at baseline, and (pm) = person-means based on measure collected across waves that was mean-averaged

Because the present study focused on how changes in SC were related to changes in mental health and well-being over time, participants were excluded if they did not provide data for at least two waves (Wrosch et al., 2018). The analyzed sample (*n* = 292) exhibited some racial diversity (71% white, 14% Black, 8% Asian, 4% two or more races, 3% other) with an average income of ~$50,000 (range $0 - $200,000) and 13 years of education on average (range 12–24). The present sample had adequate power to detect small to medium effects of the Level 1 predictors on the Level 1 outcome measures (Arend & Schafer, 2019). Attention checks were included to ensure high data quality, and previous research found that the sample was highly attentive (Hamm et al., 2022). Informed consent was obtained from all participants prior to participation, and the North Dakota State University Institutional Review Board approved all procedures and methods prior to data collection.

### Measures

**Secondary control (SC)** SC was assessed at each wave using the five-item Secondary Control Folk Beliefs Scale (Chipperfield et al., 2012; Newall et al., 2009). The scale assessed common folk beliefs that capture SC processes involving psychological acceptance (e.g., “Things will all work out in the end”) and adjustment (e.g., “I’m a believer in the idea that every cloud has a silver lining”). Participants rated their agreement using a 5-point scale (1 = *strongly disagree*, 5 = *strongly agree*). SC exhibited acceptable within-person variation, with 28% of the variance in this measure at the within-person level (ICC = 0.72). The within-person variance (σ) of SC was 0.46 and the between-person variance (τ) was 0.77. Between-person (person-mean) score were generated across waves for each participant (*M* = 3.64, *SD* = 0.91).

**Financial hardship** Consistent with previous research (Barrett, 2003; Hall et al., 2009), financial hardship was measured through reported income insufficiency, which was measured on a three-point scale (1 = *more than enough money*, 2 = *just enough money*, 3 = *not enough money*). Hardship exhibited acceptable within-person variation, with 36% of the variance in this measure at the within-person level (ICC = 0.64). The within-person variance (σ) of financial hardship was 0.41 and the between-person variance (τ) was 0.55. A mean score was computed across waves for each participant (*M* = *2.15*, *SD* = *0.69*).

**Perceived stress** Perceived stress was assessed at each wave using the 10-item Perceived Stress Scale (Cohen et al., 1983). Participants rated how frequently they experienced 10 different situations over the past week (e.g., “How often have you been upset because of something that happened unexpectedly?”). Responses were recorded using a 5-point scale (1 = *never*, 5 = *very often*). Perceived stress exhibited acceptable within-person variation (ICC = 0.78). The within-person variance (σ) of perceived stress was 0.41 and the between-person variance (τ) was 0.78. A mean score was computed across waves for each participant (*M* = 2.59, *SD* = 0.88).

**Depressive symptoms** The 10-item Center for Epidemiologic Studies Depression (CES-D10) scale was used to assess depressive symptoms at each wave (Björgvinsson et al., 2013; Radloff, 1977). Participants rated how frequently they had experienced each of 10 depressive symptoms during the past week using a four-point scale (1 = *less than one day*, 4 = *5–7 days*). Depressive symptoms exhibited acceptable within-person variation (ICC = 0.80). The within-person variance (σ) of depressive symptoms was 0.33 and the between-person variance (τ) was 0.67. A mean score was computed across waves for each participant (*M* = 1.97, *SD* = 0.75).

**Positive and negative affect** Participants indicated the extent to which they experienced six positive and six negative emotions during the past week using a 5- point scale (1 = *very slightly or not at all*, 5 = *extremely*; Hamm et al., 2015, 2016; Watson et al., 1988). Positive affect consisted of pride, excitement, happiness, satisfaction, calmness, and contentment. Negative affect consisted of anger, frustration, anxiety, loneliness, sadness, and helplessness. Positive (ICC = 0.69) and negative (ICC = 0.76) affect exhibited acceptable within-person variation. The within-person variance (σ) of positive affect was 0.53 and the between-person variance (τ) was 0.78. The within-person variance (σ) of negative affect was 0.47 and the between-person variance (τ) was 0.83. Mean scores for positive and negative affect were computed across waves for each participant (PA: *M* = 2.73, *SD* = 0.95, NA: *M* = 2.15, *SD* = 0.95).

**Life satisfaction** Life Satisfaction was assessed using Diener et al.’s 5-item satisfaction with life scale (e.g., “In most ways my life is close to my ideal”) using a 7-point scale (1 = *Strongly Disagree*, 7 = *Strongly Agree*; Diener et al., 1985). Reported life satisfaction exhibited acceptable within-person variation (ICC = 0.88). The within-person variance (σ) of life satisfaction was 0.61 and the between-person variance (τ) was 01.64. A mean score was computed across waves for each participant (*M* = 3.83, *SD* = 1.69).

**Meaning in life** Meaning was assessed using the 6-item PROMIS 6a scale, which asked participants their agreement with meaning-related items (e.g., “I experience deep meaning in life.”) using a 5-point scale (1 = *Strongly Disagree*, 5 = *Strongly Agree*; Salsman et al., 2014). Meaning in life exhibited acceptable within-person variation (ICC = 0.80). The within-person variance (σ) of meaning in life was 0.47 and the between-person variance (τ) was 0.88. As with SC, a mean score was computed across waves for each participant (*M* = 3.63, *SD* = 0.98).

**Personal growth** Growth was assessed using Ryff’s 3-item personal growth eudaimonic well-being subscale, which asked participants their agreement with items that indicated personal importance placed on growth (e.g., “I think it is important to have new experiences that challenge how you think about yourself and the world.”) using a 7-point scale (1 = *Strongly Disagree*, 7 = *Strongly Agree*; Ryff, 1989). Reported personal growth exhibited acceptable within-person variation (ICC = 0.73). The within-person variance (σ) of personal growth was 0.60 and the between-person variance (τ) was 0.98. A mean score was computed across waves for each participant (*M* = 5.53, *SD* = 1.15).

**Demographic covariates** Demographic data, including age, sex, years of education, and household income were collected at wave 1. Age in years (*M* = 46, *SD* = 16), male or female sex (1 = male, 2 = female; 52% female), years of education (13 = high school diploma, 23 = completed doctoral degree; *M* = 16.27, *SD* = 2.37), and household income in tens of thousands of dollars (*M* = 5.61, *SD* = 4.20) were reported by the participant. Table 1 shows the correlations of the variables included in the analysis.

## Results

### Rationale for analyses

We conducted two-level multilevel models (L1 = measurement occasion, L2 = individual) using the nlme package for R (Pinheiro et al., 2024) for each mental health and well-being outcome to test our hypothesis that within-person increases in SC would predict concurrent increases in well-being (Aim 1). We subsequently tested Level 1 x Level 1 interactions to examine whether within-person associations between SC and psychological well-being were strongest on occasions when people experienced increased financial hardship (Aim 2). We approached the analyses in a stepwise fashion. Step 1 involved main effect models that examined whether within-person increases in SC were related to adaptive changes in well-being. Step 2 involved interaction effect models that examined whether within-person changes in financial hardship moderated the association between changes in SC and changes in well-being. All models controlled for time as well as baseline differences in age, education, sex, and income.

### Step 1: main effect models

Multilevel models tested the associations between SC and the mental health and well-being outcomes when controlling for linear changes over time and between-person differences in SC and demographic variables. Level 1 (within-person) predictor variables included an intercept, as well as person-mean centered: time in study, SC, and financial hardship. Level 1 models were constructed as follows, where WB = well-being outcomes, i = occasions, and j = individuals:

$$WB_{{ij}} = \beta _{{0j}} + \beta _{{1j}} (Time_{{ij}} ){\text{ }} + \beta _{{2j}} (SC_{{ij}} ){\text{ }} + \beta _{{3j}} \left( {Hardship_{{ij}} } \right) + {\text{ }}e_{{ij}}$$

Level 2 (between-person) predictor variables included individual differences in average levels of SC across the study, as well as baseline age, sex, education, income, and financial hardship. All Level 2 predictors were grand-mean centered. Level 2 models were constructed as follows:

$$ \begin{aligned} \beta _{{0j}} = & \gamma _{{00}} + \gamma _{{01}} (Age_{{.j}} ){\text{ }} + \gamma _{{02}} (Sex_{{.j}} ){\text{ }} + \gamma _{{03}} (Edu_{{.j}} ){\text{ }} \\ & + \gamma _{{04}} (Inc_{{.j}} ){\text{ }} + \gamma _{{06}} (SC_{{.j}} ){\text{ }} + \gamma _{{05}} (Hardship_{{.j}} ) + {\text{ }}U_{{0j}} \\ \end{aligned} $$

$$\beta _{{1j}} = \gamma _{{10}} + {\text{ }}U_{{1j}}$$

$$\beta _{{2j}} = \gamma _{{20}} + {\text{ }}U_{{2j}}$$

$$\beta _{{3j}} = \gamma _{{30}} + {\text{ }}U_{{3j}}$$

Results showed that SC, at both the within- and between-person levels, were significant predictors of depressive symptoms, life satisfaction, meaning in life, negative affect, positive affect, person growth, and perceived stress in adaptive directions (see Table 2). Increases in within-person SC were associated with corresponding increases in life satisfaction, meaning in life, positive affect, and personal growth. Increases in SC also related to decreases in depressive symptoms, negative affect, and perceived stress. As expected from prior research, this pattern was replicated for between-person differences in SC. The within-person findings indicate that, when individuals reported more SC than their average levels, they also tended to experience more adaptive mental health and well-being than they typically did across the 2-year study timespan. Within-person increases in financial hardship indicated that when individuals felt that their income was less sufficient to meet their needs than usual, they experienced declines in well-being. Between-person differences in financial hardship were also linked to higher levels of depressive symptoms and perceived stress and lower levels of life satisfaction, meaning in life, and positive affect (Table 3).

**Table 2:** Main effects of secondary control and financial hardship on Well-Being

Variable	PS γ (SE)	DP γ (SE)	PA γ (SE)	NA γ (SE)	LS γ (SE)	MN γ (SE)	PG γ (SE)
Level-1 (wp)							
Intercept	2.60 (0.04)**	1.97 (0.03)**	2.74 (0.04)**	2.15 (0.04)**	3.82 (0.08)**	3.64 (0.04)**	5.53 (0.05)**
Time	−0.01 (0.00)**	−0.01 (0.00)**	0.01 (0.00)**	−0.01 (0.00)**	0.00 (0.00)	0.00 (0.00)*	−0.00 (0.00)
SC	−0.14 (0.03)**	−0.10 (0.03)**	0.21 (0.04)**	−0.11 (0.04)**	0.12 (0.05)*	0.17 (0.03)**	0.23 (0.05)**
Hardship	0.08 (0.03)*	0.05 (0.03)*	−0.10 (0.04)*	0.08 (0.04)*	−0.15 (0.05)**	−0.01 (0.03)	0.02 (0.05)
Level-2 (bp)							
Age	−0.01 (0.00)**	−0.01 (0.00)**	0.00 (0.00)	−0.01 (0.00)**	0.00 (0.01)	0.00 (0.00)	−0.01 (0.00)**
Sex	0.31 (0.08)**	0.22 (0.07)**	−0.30 (0.08)**	0.32 (0.09)**	−0.23 (0.16)	−0.07 (0.09)	0.22 (0.11)*
Edu	−0.02 (0.02)	−0.02 (0.01)	0.03 (0.02)	−0.02 (0.02)	0.12 (0.02)**	0.03 (0.02)	0.04 (0.02)
Inc	−0.00 (0.01)	−0.00 (0.01)	0.01 (0.01)	−0.01 (0.01)	0.04 (0.02)	0.01 (0.01)	0.01 (0.01)
SC	−0.37 (0.05)**	−0.32 (0.04)**	0.45 (0.05)**	−0.34 (0.05)**	0.87 (0.10)**	0.60 (0.05)**	0.62 (0.05)**
Hardship	0.47 (0.07)**	0.32 (0.06)**	−0.33 (0.08)**	0.44 (0.08)**	−0.94 (0.15)**	−0.24 (0.08)**	−0.16 (0.10)

*wp *within-person, *bp *between-person, *PS *perceived stress, *DP* depressive symptoms, *PA *positive affect, *NA *negative affect, *LS *life satisfaction, *MN *meaning in life, *PG *personal growth, *Edu* education, and *Inc *income. Within-person variables were person-mean centered, and between-person variables were grand-mean centered. Within-person SC was significantly related to all measured well-being variables. Parameter estimates for the main predictor variable (within-person SC) are shown in bold font

*p < 0.05, **p < 0.005

**Table 3:** Moderated effects of secondary control on Well-Being as a function of financial hardship

Variable	PS (SE)	DP (SE)	PA (SE)	NA (SE)	LS (SE)	MN (SE)	PG (SE)
Level-1 (wp)							
Intercept	2.61 (0.04)**	1.98 (0.03)**	2.74 (0.04)**	2.16 (0.04)**	3.83 (0.08)**	3.63 (0.04)**	5.53 (0.05)**
Time	0.00 (0.00)**	−0.01 (0.00)**	0.01 (0.00)**	−0.01 (0.00)**	0.00 (0.00)	0.00 (0.00)**	0.00 (0.00)
SC	−0.13 (0.03)**	−0.11 (0.03)**	0.21 (0.04)**	−0.12 (0.04)**	0.15 (0.05)*	0.19 (0.04)**	0.22 (0.05)**
Hardship	0.11 (0.03)**	0.06 (0.03)*	−0.11 (0.04)*	0.09 (0.04)*	−0.13 (0.05)*	−0.02 (0.04)	0.03 (0.05)
SC x Hardship	−0.24 (0.09)*	**−0.08 (0.07)**	**0.06 (0.12)**	**−0.16 (0.10)**	**0.10 (0.14)**	**−0.14 (0.10)**	0.35 (0.14)*
Level-2 (bp)							
Age	−0.01 (0.00)**	−0.01 (0.00)**	0.00 (0.00)	−0.01 (0.00)**	0.00 (0.01)	0.00 (0.00)	−0.01 (0.00)**
Sex	0.33 (0.08)**	0.24 (0.07)**	−0.30 (0.08)**	0.37 (0.09)**	−0.23 (0.16)	−0.07 (0.09)	0.20 (0.10)*
Edu	−0.02 (0.02)	−0.02 (0.01)	0.03 (0.02)	−0.02 (0.02)	0.12 (0.03)**	0.03 (0.02)	0.03 (0.02)
Inc	0.00 (0.01)	0.01 (0.01)	0.00 (0.01)	0.00 (0.01)	0.04 (0.02)	0.01 (0.01)	0.02 (0.02)
SC	−0.36 (0.05)**	−0.32 (0.04)**	0.43 (0.05)**	−0.35 (0.05)**	0.89 (0.10)**	0.60 (0.05)**	0.61 (0.06)**
Hardship	0.46 (0.07)**	0.33 (0.06)**	−0.32 (0.08)**	−0.16 (0.10)	−0.91 (0.15)**	−0.24 (0.08)**	−0.14 (0.10)
SC x Hardship	−0.04 (0.07)	−0.04 (0.07)	0.07 (0.08)	−0.01 (0.08)	−0.01 (0.15)	0.08 (0.08)	0.08 (0.10)

*wp *within-person, *bp *between-person, *DP *depressive symptoms, *LS *life satisfaction, *NA* negative affect, *PA *positive affect, *PG *personal growth, *PS *perceived stress, *Edu *education, and *Inc *income. Within-person variables were person-mean centered, between-person variables were grand-mean centered. The interaction between within-person secondary control within-person hardship was significant for personal growth and perceived stress. Parameter estimates for the interaction of primary interest (within-person SC x within-person hardship) are shown in bold font

*p < 0.05, **p < 0.005

### Step 2: moderation models

Interaction models were conducted to test whether within-person changes in financial hardship moderated the link between within-person SC and changes in the well-being outcomes. The models were specified as in Step 1. The only difference was the addition of a Level 1 SC x hardship and Level 2 SC x hardship interaction terms. As in Step 1, all Level 1 variables were person-mean centered and all Level 2 variables were grand-mean centered. The Level 1 models were specified as follows:

$$ \begin{aligned} WB_{{ij}} = & \beta _{{0j}} + \beta _{{1j}} (SC_{{ij}} ){\text{ }} + \beta _{{2j}} (Time_{{ij}} ){\text{ }} + \beta _{{3j}} (Hardship_{{ij}} ) \\ & {\text{ }} + \beta _{{4j}} ((SC_{{ij}} )*{\text{ }}(Hardship_{{ij}} )){\text{ }} + {\text{ }}e_{{ij}} \\ \end{aligned} $$

Level 2 models were specified as follows:

$$\begin{aligned} \beta _{{0j}} = & \gamma _{{00}} + \gamma _{{01}} (Age_{{.j}} ){\text{ }} + \gamma _{{02}} (Sex_{{.j}} ){\text{ }} + \gamma _{{03}} (Edu_{{.j}} ){\text{ }} + \gamma _{{04}} (Inc_{{.j}} ) \\ & {\text{ }} + \gamma _{{05}} (Hardship_{{.j}} ){\text{ }} + \gamma _{{06}} (SC_{{.j}} ){\text{ }} + \gamma _{{07}} (\left( {SC_{{.j}} } \right){\text{ }}*(Hardship_{{.j}} )) + U_{{0j}} \\ \end{aligned}$$

$$\beta _{{1j}} = \gamma _{{10}} + {\text{ }}U_{{1j}}$$

$$\beta _{{2j}} = \gamma _{{20}} + {\text{ }}U_{{2j}}$$

$$\beta _{{3j}} = \gamma _{{30}} + {\text{ }}U_{{3j}}$$

$$\beta _{{4j}} = \gamma _{{40}} + {\text{ }}U_{{4j}}$$

**Fig. 1:**
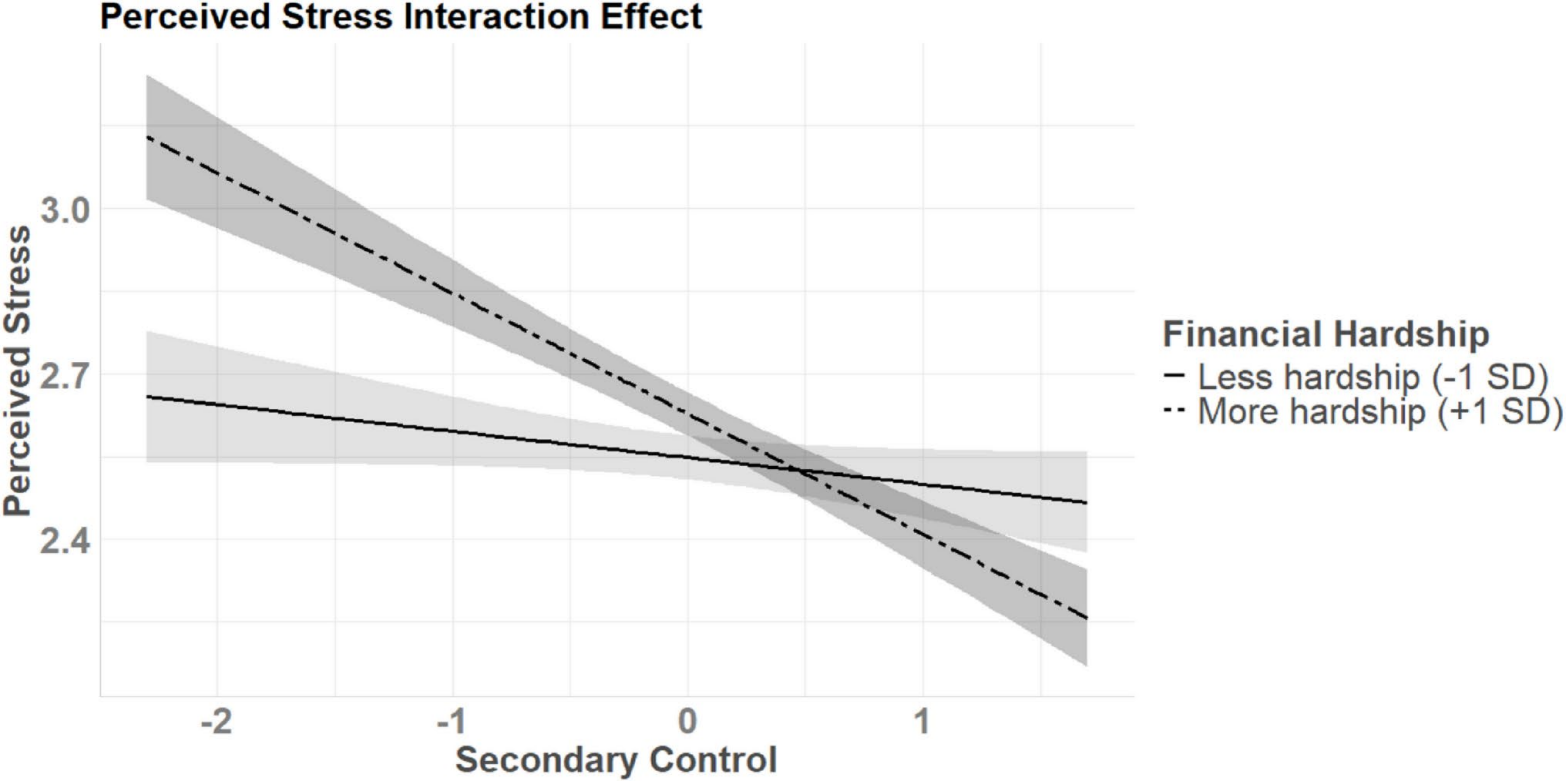
The within-person relationship between secondary control and perceived stress, moderated by financial hardship. This graph depicts the within-person relationship between changes in secondary control and perceived stress when the participant is experiencing less financial hardship (darker, dotted) or greater financial hardship than usual (lighter, straight). Ribbons indicate +/- 1SE

**Fig. 2:**
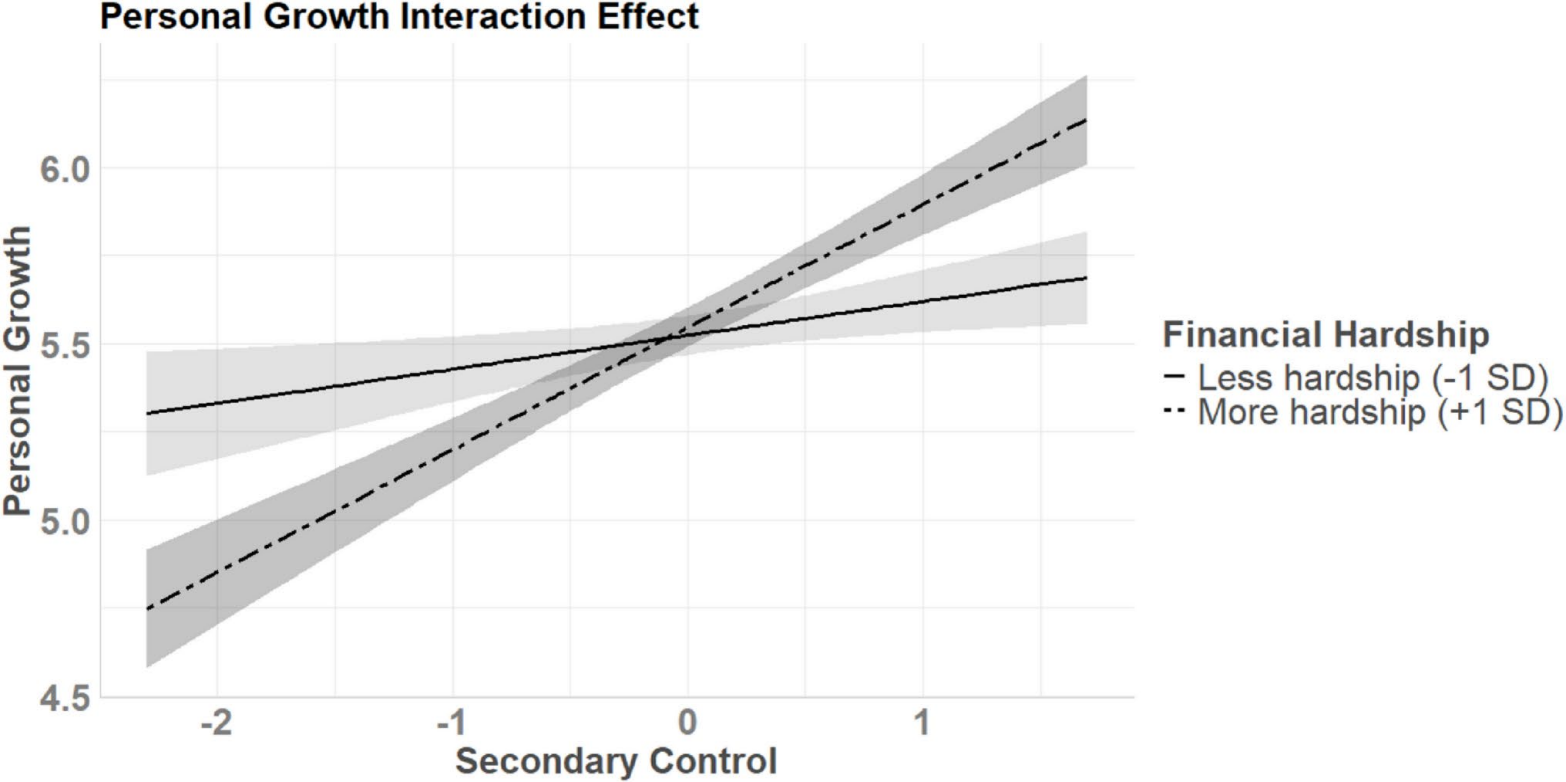
The within-person relationship between secondary control and personal growth, moderated by financial hardship. This graph depicts the within-person relationship between changes in secondary control and personal growth when the participant is experiencing less financial hardship (darker, dotted) or greater financial hardship than usual (lighter, straight). Ribbons indicate +/- 1SE

Partially consistent with the hypotheses for Aim 2, significant L1 SC x L1 Financial Hardship interactions were observed for perceived stress and personal growth, such that increases in SC had a pronounced association with both outcomes in circumstances under which individuals reported more financial hardship than usual. Interactions were not observed for the other outcome measures. Significant interactions were probed using simple slopes at low (−1 SD) and high (+ 1 SD) values of financial hardship. On occasions when individuals experienced more hardship than usual, increases in SC were strongly linked corresponding decreases in perceived stress (γ = − 0.22, *SE* = 0.046, *p* < 0.001; pseudo-standardized: γ = −0.22). On occasions when individuals experienced less hardship than usual, increases in SC had no relationship with changes in perceived stress (*γ* = − 0.05, *SE* = 0.048, *p* = 0.315; pseudo-standardized: γ = −0.05). A similar pattern was observed for personal growth. On occasions when individuals experienced more hardship than usual, increases in SC strongly were strongly linked to increases in personal growth (*γ* = 0.35, *SE* = 0.068, *p* < 0.001; pseudo-standardized: γ = 0.24). In contrast, on occasions when individuals experienced less hardship than usual, increases in SC were unrelated to changes in personal growth (*γ* = 0.10, *SE* = 0.072, *p* = 0.171; pseudo-standardized: γ = 0.07). These relationships can be seen in Figs. 1 and 2, respectively.

These interactions were also probed with hardship as the predictor and SC as the moderator to examine how increases in SC may buffer against declines in well-being that may otherwise occur in response to increased financial hardships. A buffering pattern was observed for perceived stress but not for personal growth. On occasions when individuals reported more SC than usual, increases in hardship were unrelated to changes in perceived stress (*γ* = 0.01, *SE* = 0.050, *p* = 0.809). In contrast, when individuals reported less SC than usual, increases in hardship were strongly linked to corresponding increases in perceived stress (*γ* = 0.21, *SE* = 0.050, *p* < 0.001). On occasions when individuals reported more SC than usual, increases in hardship were more strongly linked to increases in personal growth (*γ* = 0.18, *SE* = 0.078, *p* = 0.024). In contrast, when individuals reported less SC than usual, increases in hardship were unrelated to changes in personal growth (*γ* = −0.11, *SE* = 0.077, *p* = 0.149).

### Supplemental analyses

**Age moderation** Due to prior research that suggests differing salience and benefits of SC across the adult lifespan, a supplemental analysis examined a 3-way, cross-level interaction between within-person SC, within-person hardship, and between-person age on the well-being outcomes (Haynes et al., 2009; Heckhausen et al., 2010; Schulz & Heckhausen, 1996). The models were specified as described in the moderated effects section, with the addition of the interaction between L1 SC, L1 financial hardship, and L2 baseline age (as well as all lower-order interactions between these variables). 3-way interactions were observed for both negative and positive affect, but not the other well-being outcomes. The pattern of moderated effects suggested that the relationship between SC and both measures of affect were further moderated by age. The pattern we found suggests that the benefits of SC on positive and negative affect during periods of financial hardship were more pronounced for older, as opposed to younger, adults. Conversely, younger adults benefitted more from SC when they were experiencing less hardship than they typically did. See the supplemental materials for a more detailed summary of the age moderated findings.

**Primary control** Due to its theoretical and conceptual relevance in the context of SC, supplemental analyses were conducted to determine whether results were consistent when controlling for Level-1 and Level-2 primary control beliefs. Results showed that the main effects of Level-1 SC on all well-being outcomes remained significant, with the sole exceptions of life satisfaction and negative affect. The L1 SC x L1 Hardship interaction effects on perceived stress and personal growth both remained significant.

## Discussion

Our study assessed how changes in SC were associated with corresponding shifts in mental health and well-being over time during COVID-19 and how this relationship may be affected by fluctuations in one’s ability to pay for their basic necessities. Findings showed that shifts in SC were adaptive, such that increases in SC compared to an individual’s average level were linked to corresponding increases in life satisfaction, meaning in life, positive affect, and personal growth, and decreases in depressive symptoms, negative affect, and perceived stress. Financial hardship moderated this relationship for perceived stress and personal growth, such that the benefits of increasing SC were pronounced on occasions when people reported greater hardship than usual. These findings advance the literature by providing evidence that experiencing greater SC than one’s typical level may serve to protect well-being, especially during periods of greater financial hardship. Thus, increasing SC may provide a means of alleviating the negative impacts of fluctuating financial security. This research also informs theories of control by providing new evidence for the dynamic role of SC in response to rapid changes in the controllability of life circumstances.

### Coupled changes in secondary control and well-being during COVID-19

Consistent with Aim 1 of the study, within-person fluctuations in SC were associated with changes in all well-being measures. Positive affect, life satisfaction, meaning in life, and personal growth generally increased when participants felt higher SC than they normally did in the context of the pandemic. The reverse was true for perceived stress, depressive symptoms, and negative affect, which tended to be reduced when participants had higher than usual SC.

These findings are in line with previous research on between-person (individual differences) in SC that indicates the general positive link between SC and mental health and well-being (Chipperfield et al., 2017; Swift & Chipperfield, 2013; Toyama, 2022). Our findings extend prior research by showing that the between-person associations are consistent at the within-person level, such that increases in SC can lead to corresponding adaptive shifts in well-being. This represents a more dynamic view of SC, not just as an unchanging (static) characteristic of an individual, but also as a shifting component of one’s psychosocial state. This is partially reflected in the ICC of SC, which was 0.72; this indicates that 28% of the changes in SC can be attributed to dynamic, within-person fluctuations over time. On an individual level, this indicates that increases in SC may lead to meaningful changes in well-being. Conversely, this also means that reductions in SC could have negative consequences for well-being. In the context of COVID-19, this research provides evidence for the importance of maintaining internal (secondary) control in otherwise uncontrollable circumstances.

We also found evidence that increases in financial hardship were related to detrimental changes in well-being. Within-person increases in hardship—when an individual felt less able to pay for their needs than usual—were related to maladaptive changes in perceived stress, depressive symptoms, positive affect, negative affect, and life satisfaction. Similar patterns were found in between-person differences in hardship. This provides support for the notion that financial hardship reflects a dynamic contextual circumstance with potentially detrimental impacts for well-being.

### The moderating role of dynamic changes in financial hardship

In line with Aim 2 of the study, the within-person relationship between SC and select well-being outcomes depended on dynamic changes in financial hardship. The interaction between within-person SC and within-person financial hardship was significant for perceived stress and personal growth. When individuals experienced greater financial hardship than usual, the link between increases in SC and adaptive changes in personal growth and perceived stress were pronounced. This is a novel finding that points to the importance of SC in the context of dynamic shifts in socioeconomic circumstances. Not only is SC beneficial in the context of longer-term age-related changes or between different groups of people who experience systemic inequalities, but also over briefer timescales that involve significant within-person changes or fluctuations in financial hardship. This also points to the relevance of socioeconomic hardship as veridical indicator of low control environments in which SC may be most beneficial, consistent with Chen and Miller’s work (Chen & Miller, 2012; Chen et al., 2013, 2015).

The significant moderation findings for only perceived stress and personal growth denote a potentially interesting connection between these variables, SC, and financial hardship. It is possible that the non-significant findings could be potentially due to the study being underpowered. All interactions trended in the same direction as personal growth and perceived stress, such that within-person increases in SC were more tightly (albeit not significantly) coupled with increased well-being and mental health during periods of heightened financial hardship. However, there could also be meaningful differences in how SC and hardship are specifically linked to personal growth and perceived stress. Perceived stress could be more directly linked to immediate financial difficulties, amplifying the relationship between fluctuations in SC and stress during periods of financial difficulty. Personal growth showed an interesting relationship such that participants reported the highest levels of personal growth on occasions that where they experienced increased SC *and* increased financial hardship (rather than higher SC simply buffering an adverse impact of increased hardship). This potentially indicates that a combination of SC and financial hardship contributes more to a person’s perceived personal growth, real or not. These findings could indicate actual growth, where going through financial hardship and still feeling an internal sense of control could lead a person to make more meaningful changes to their understanding of the world. This could also indicate a sort of “comforting lie”, where people may tend to justify their suffering as an indicator of growth rather than outright hardship.

### SC, hardship, and well-being across the adult lifespan

The supplemental age moderation analyses revealed a potentially more complex relationship between SC and select measures of well-being. Specifically, the supplemental analyses indicated that the moderated relationship between within-person SC and financial hardship on hedonic well-being (positive and negative affect) depended on whether participants were younger vs. older adults. Results suggest that, as a person grows older, the link between changes in SC and corresponding shifts in hedonic well-being may be strongest when an individual experiences difficult circumstances (increased hardship).

These findings are broadly in line with theories of lifespan control and emotional aging that point to (a) the increased salience and adaptive value of SC as individuals age and (b) the greater prioritization of, and capacity to maintain, affective well-being in older age (Carstensen, 1995; Heckhausen et al., 2010; Kunzman, 2014). That is, our supplemental findings suggest that secondary control may be more beneficial for well-being in younger adulthood during typical circumstances (i.e., daily life in younger adulthood may be sufficiently stressful to derive affective benefits from secondary control without additional stressors such as financial hardship). However, secondary control may only become beneficial in older age when circumstances are more stressful than usual (i.e., older adulthood alone may not be sufficiently stressful to elicit the affective benefits of secondary control in the absence of additional stressors).

### Strengths, limitations, and future directions

The present research has several strengths. The use of data from a nationally representative U.S. sample aged 18–80 years allow for more generalizability than a convenience sample. The longitudinal nature of the study involved five assessments of the predictor, moderator, and outcome variables, providing a unique opportunity to examine the dynamic interplay between SC, hardship, and well-being over a 2-year period. The context of the COVID-19 pandemic may have also contributed to rapid changes in individual circumstances that enabled our examination of the relationships between within-person changes in SC, financial hardship, and well-being.

However, there are several limitations of the data. First, the timescale of the collected data is over month- and year-long interval periods, such that the data could not be used to examine changes that are likely to occur over shorter time scales. Future research could extend this work by examining whether more micro-level, day-to-day fluctuations in SC may have an impact on mental health and well-being. Second, our approach focused on the role of concurrent or coupled changes in SC and well-being as opposed to prospective or lagged effects of SC on subsequent changes in well-being. We adopted a coupled change approach rather than a time-lagged approach because our interest was in how the dynamic relationship between SC and well-being unfolds concurrently in response to present financial hardship (rather than how changes in SC months or years ago predicts current well-being). Our focus on concurrent changes was also due in part to the present study design which was not well-suited to a time-lagged approach given that the time between waves changed across the study period (initial waves were separated by approximately a month and the final waves separated by approximately a year; see Hamm, Barlow, et al., 2023a). Third, although the sample was nationally representative of U.S. adults, it was predominantly made up of white individuals, limiting its generalizability across racial and ethnic groups. Future research is needed to examine the within-person relationships between SC and well-being in non-white samples. Finally, the data collected was all self-reported. Potential future research could examine behavioral measures of health and well-being, such as sleep or physical activity, to better determine the impact of SC on health- and well-being-related outcomes.

## Conclusion

In conclusion, this study found that within-person increases in SC predicted concurrent increases in central indicators of mental health, hedonic well-being, and eudaimonic well-being. We also found that this relationship was moderated by financial hardship in the case of perceived stress and personal growth. These findings provide new evidence for the dynamic role of SC at the within-person level, and point to financial hardship as a potential circumstance of interest in the context of SC. Our research indicates that increases in daily SC may be linked to improved daily well-being, and that these improvements may be more acutely felt when individuals are undergoing losses of (external) control in the form of increased financial hardship. In sum, the present study provides new evidence that there may be dynamic benefits to “finding the silver lining” in day-to-day life.

## Supplementary Information

Below is the link to the electronic supplementary material.

Supplementary Material 1

## Data Availability

Deidentified study data is available from the authors on request.

## References

[CR1] Arend, M. G., & Schäfer, T. (2019). Statistical power in two-level models: A tutorial based on Monte Carlo Simulation. *Psychological Methods,**24*(1), 1–19. 10.1037/met0000195 30265048 10.1037/met0000195

[CR2] Barrett, A. E. (2003). Socioeconomic status and age identity: The role of dimensions of health in the subjective construction of age. *The Journals of Gerontology Series B: Psychological Sciences and Social Sciences*. 10.1093/geronb/58.2.s101 12646599 10.1093/geronb/58.2.s101

[CR3] Björgvinsson, T., Kertz, S. J., Bigda-Peyton, J. S., McCoy, K. L., & Aderka, I. M. (2013). Psychometric properties of the CES-D-10 in a psychiatric sample. *Assessment,**20*(4), 429–436. 10.1177/1073191113481998 23513010 10.1177/1073191113481998

[CR4] Butterworth, P., Olesen, S. C., & Leach, L. S. (2012). The role of hardship in the association between socio-economic position and depression. *Australian & New Zealand Journal of Psychiatry,**46*(4), 364–373. 10.1177/0004867411433215 22508596 10.1177/0004867411433215

[CR5] Carstensen, L. L. (1995). Evidence for a life-span theory of socioemotional selectivity. *Current Directions in Psychological Science,**4*(5), 151–156. 10.1111/1467-8721.ep11512261 10.1177/09637214211011468 PMC8340497 34366582

[CR7] Chen, E., Lee, W. K., Cavey, L., & Ho, A. (2013). Role models and the psychological characteristics that buffer low-socioeconomic‐status youth from cardiovascular risk. *Child Development,**84*(4), 1241–1252. 10.1111/cdev.12037 23278857 10.1111/cdev.12037

[CR8] Chen, E., McLean, K. C., & Miller, G. E. (2015). Shift-and-persist strategies. *Psychosomatic Medicine*, *77*(3), 371–382. 10.1097/psy.0000000000000157 26167560 10.1097/psy.0000000000000157 PMC5890430

[CR6] Chen, E., & Miller, G. E. (2012). shift-and-persist strategies. *Perspectives on Psychological Science*, *7*(2), 135–158. 10.1177/1745691612436694 23144651 10.1177/1745691612436694 PMC3491986

[CR10] Chipperfield, J. G., Hamm, J. M., Perry, R. P., & Ruthig, J. C. (2017). Perspectives on studying perceived control in the twenty-first century. *The Happy Mind: Cognitive Contributions to Well-Being*. 10.1007/978-3-319-58763-9_12

[CR9] Chipperfield, J. G., & Perry, R. P. (2006). Primary- and secondary-control strategies in later life: Predicting hospital outcomes in men and women. *Health Psychology,**25*(2), 226–236. 10.1037/0278-6133.25.2.226 16569115 10.1037/0278-6133.25.2.226

[CR12] Chipperfield, J. G., Perry, R. P., Bailis, D. S., Ruthig, J. C., & Chuchmach loring, P. (2007). Gender differences in use of primary and secondary control strategies in older adults with major health problems. *Psychology & Health,**22*(1), 83–105. 10.1080/14768320500537563

[CR11] Chipperfield, J. G., Perry, R. P., & Stewart, T. L. (2012). Perceived control. *Encyclopedia of Human Behavior*. 10.1016/b978-0-12-375000-6.00109-9

[CR13] Cohen, S., Kamarck, T., & Mermelstein, R. (1983). A global measure of perceived stress. *Journal of Health and Social Behavior*, *24*(4), 385. 10.2307/2136404 6668417

[CR14] Credé, M., & Niehorster, S. (2011). Adjustment to college as measured by the student adaptation to college questionnaire: A quantitative review of its structure and relationships with correlates and consequences. *Educational Psychology Review*, *24*(1), 133–165. 10.1007/s10648-011-9184-5

[CR15] Diener, E., Emmons, R. A., Larsen, R. J., & Griffin, S. (1985). The satisfaction with life scale. *Journal of Personality Assessment,**49*, 71–75. 16367493 10.1207/s15327752jpa4901_13

[CR16] Fisher, J. R., Tran, T. D., Hammarberg, K., Sastry, J., Nguyen, H., Rowe, H., Popplestone, S., Stocker, R., Stubber, C., & Kirkman, M. (2020). Mental health of people in Australia in the first month of COVID-19 restrictions: A national survey. *Medical Journal of Australia,**213*(10), 458–464. 10.5694/mja2.50831 33107063 10.5694/mja2.50831 PMC7756394

[CR17] Fisher, R. S., Sharp, K. M., Prussien, K. V., Himelhoch, A. C., Murphy, L. K., Rodriguez, E. M., Young-Saleme, T. K., Vannatta, K., Compas, B. E., & Gerhardt, C. A. (2021). Coping trajectories and the health-related quality of life of childhood cancer survivors. *Journal of Pediatric Psychology,**46*(8), 960–969. 10.1093/jpepsy/jsab017 33738496 10.1093/jpepsy/jsab017 PMC8376259

[CR18] Frankham, C., Richardson, T., & Maguire, N. (2020). Psychological factors associated with financial hardship and mental health: A systematic review. *Clinical Psychology Review,**77*, Article 101832. 10.1016/j.cpr.2020.101832 32088498 10.1016/j.cpr.2020.101832

[CR19] Gallo, L. C., & Matthews, K. A. (2003). Understanding the association between socioeconomic status and physical health: Do negative emotions play a role? *Psychological Bulletin,**129*(1), 10–51. 10.1037/0033-2909.129.1.10 12555793 10.1037/0033-2909.129.1.10

[CR20] Hall, M. H., Matthews, K. A., Kravitz, H. M., Gold, E. B., Buysse, D. J., Bromberger, J. T., Owens, J. F., & MaryFran, S. (2009). Race and financial strain are independent correlates of sleep in midlife women: The SWAN sleep study. *Sleep,**32*(1), 73–82. 10.5665/sleep/32.1.73 19189781 PMC2625326

[CR21] Hamm, J. M., Barlow, M. A., Garcia, O. G., & Duggan, K. A. (2023a). Context-dependent shifts in self‐regulatory personality processes during COVID‐19: Changes in control predict dynamic shifts in goal reengagement capacity. *Social and Personality Psychology Compass*. 10.1111/spc3.12771 39399317 10.1111/spc3.12771 PMC11469646

[CR22] Hamm, J. M., Lachman, M. E., Duggan, K. A., Mogle, J. A., McGrath, R., Parker, K. P., & Klepacz, L. M. (2025). When and how perceived control buffers against cognitive declines: A moderated mediation analysis. *Psychology and Aging,**40*, 39–53. 39133611 10.1037/pag0000841 PMC11781980

[CR23] Hamm, J. M., Perry, R. P., Chipperfield, J. G., Heckhausen, J., & Parker, P. C. (2016). A motivation-enhancing treatment to sustain goal engagement during life course transitions. *Motivation and Emotion,**40*(6), 814–829. 10.1007/s11031-016-9576-4

[CR24] Hamm, J. M., Perry, R. P., Chipperfield, J. G., Stewart, T. L., & Heckhausen, J. (2015). Motivation-focused thinking: Buffering against stress-related physical symptoms and depressive symptomology. *Psychology & Health,**30*(11), 1326–1345. 10.1080/08870446.2015.1050394 25978418 10.1080/08870446.2015.1050394

[CR25] Hamm, J. M., Shane, J., Pierce, M. J., & Duggan, K. A. (2023b). Individual differences in patterns of developmental opportunity and constraint during COVID‐19: Implications for longitudinal well‐being. *Social and Personality Psychology Compass*. 10.1111/spc3.12788 39399318 10.1111/spc3.12788 PMC11469641

[CR61] Hamm, J. M., Tan, J. X., Barlow, M. A., Delaney, R. L., & Duggan, K. A. (2022). Goal adjustment capacities in uncontrollable life circumstances: Benefits for psychological well-being during COVID-19. Motivation and Emotion, 46(3), 319–335. 10.1007/s11031-022-09941-6 10.1007/s11031-022-09941-6 PMC9124288 35633867

[CR26] Haynes, T. L., Heckhausen, J., Chipperfield, J. G., Perry, R. P., & Newall, N. E. (2009). Primary and secondary control strategies: Implications for health and well-being among older adults. *Journal of Social and Clinical Psychology,**28*(2), 165–197. 10.1521/jscp.2009.28.2.165

[CR27] Heckhausen, J., & Schulz, R. (1995). A life-span theory of control. *Psychological Review*, *102*(2), 284–304. 10.1037/0033-295x.102.2.284 7740091 10.1037/0033-295x.102.2.284

[CR28] Heckhausen, J., Wrosch, C., & Schulz, R. (2010). A motivational theory of life-span development. *Psychological Review*, *117*(1), 32–60. 10.1037/a0017668 20063963 10.1037/a0017668 PMC2820305

[CR29] Heckhausen, J., Wrosch, C., & Schulz, R. (2019). Agency and motivation in adulthood and old age. *Annual Review of Psychology*, *70*(1), 191–217. 10.1146/annurev-psych-010418-103043 30110574 10.1146/annurev-psych-010418-103043

[CR30] Helzer, E. G., & Jayawickreme, E. (2015). Control and the “good life.” *Social Psychological and Personality Science,**6*(6), 653–660. 10.1177/1948550615576210

[CR31] Kunzmann, U., Kappes, C., & Wrosch, C. (2014). Emotional aging: A discrete emotions perspective. *Frontiers in Psychology*. 10.3389/fpsyg.2014.00380 24834060 10.3389/fpsyg.2014.00380 PMC4018521

[CR32] Lachman, M. E. (2006). Perceived control over aging-related declines. *Current Directions in Psychological Science,**15*(6), 282–286. 10.1111/j.1467-8721.2006.00453.x

[CR34] Lahelma, E., Laaksonen, M., Martikainen, P., Rahkonen, O., & Sarlio-Lähteenkorva, S. (2006). Multiple measures of socioeconomic circumstances and common mental disorders. *Social Science & Medicine*, *63*(5), 1383–1399. 10.1016/j.socscimed.2006.03.027 16690186 10.1016/j.socscimed.2006.03.027

[CR35] Matthews, K. A., & Gallo, L. C. (2011). Psychological perspectives on pathways linking socioeconomic status and physical health. *Annual Review of Psychology,**62*(1), 501–530. 10.1146/annurev.psych.031809.130711 20636127 10.1146/annurev.psych.031809.130711 PMC3121154

[CR36] Morling, B., & Evered, S. (2006). Secondary control reviewed and defined. *Psychological Bulletin*, *132*(2), 269–296. 10.1037/0033-2909.132.2.269 16536644 10.1037/0033-2909.132.2.269

[CR37] Newall, N. E., Chipperfield, J. G., Daniels, L. M., Hladkyj, S., & Perry, R. P. (2009). Regret in later life: Exploring relationships between regret frequency, secondary interpretive control beliefs, and health in older individuals. *The International Journal of Aging and Human Development,**68*(4), 261–288. 10.2190/ag.68.4.a 19711617 10.2190/AG.68.4.a

[CR38] Nikolaidis, A., DeRosa, J., Kass, M., Droney, I., Alexander, L., Di Martino, A., Bromet, E., Merikangas, K., Milham, M. P., & Paksarian, D. (2022). Heterogeneity in COVID-19 pandemic-induced lifestyle stressors predicts future mental health in adults and children in the US and UK. *Journal of Psychiatric Research,**147*, 291–300. 10.1016/j.jpsychires.2021.12.058 35123338 10.1016/j.jpsychires.2021.12.058 PMC8720815

[CR39] Parker, P. C., Perry, R. P., Chipperfield, J. G., Hamm, J. M., Daniels, L. M., & Dryden, R. P. (2022). Adjustment and acceptance beliefs in achievement settings: Implications for student wellbeing. *Social Psychology of Education,**25*(5), 1031–1049. 10.1007/s11218-022-09717-3 35996464 10.1007/s11218-022-09717-3 PMC9385084

[CR40] Pinheiro, J., Bates, D., & Core Team, R. (2024). _nlme: Linear and Nonlinear Mixed Effects Models_. R package version 3.1–166. https://CRAN.R-project.org/package=nlme

[CR41] Prati, G., & Mancini, A. D. (2021). The psychological impact of COVID-19 pandemic lockdowns: A review and meta-analysis of longitudinal studies and natural experiments. *Psychological Medicine,**51*(2), 201–211. 10.1017/s0033291721000015 33436130 10.1017/S0033291721000015 PMC7844215

[CR42] Prihadi, K., Tan, C. Y. H., Tan, R. T. S., Ling Yong, P., Yong, J. H. E., Tinagaran, S., & Yeow, J. L. H. (2018). Procrastination and learned-helplessness among university students: The mediation effect of internal locus of control. *Electronic Journal of Research in Educational Psychology,**16*(46), 579–595. 10.25115/ejrep.v16i46.2236

[CR43] Radloff, L. S. (1977). The CES-D scale. *Applied Psychological Measurement,**1*(3), 385–401. 10.1177/014662167700100306

[CR44] Robinson, S. A., & Lachman, M. E. (2020). Daily control beliefs and cognition: The mediating role of physical activity. *The Journals of Gerontology. Series B, Psychological Sciences and Social Sciences,**75*(4), 772–782. 10.1093/geronb/gby081 29982815 10.1093/geronb/gby081 PMC7328023

[CR45] Rothbaum, F., Weisz, J. R., & Snyder, S. S. (1982). Changing the world and changing the self: A two-process model of perceived control. *Journal of Personality and Social Psychology*, *42*(1), 5–37. 10.1037/0022-3514.42.1.5

[CR46] Rutter, M. (2007). Proceeding from observed correlation to causal inference: The use of natural experiments. *Perspectives on Psychological Science*, *2*(4), 377–395. 10.1111/j.1745-6916.2007.00050.x 26151974 10.1111/j.1745-6916.2007.00050.x

[CR47] Ryff, C. (1989). Happiness is everything, or is it? Explorations on the meaning of psychological well-being. *Journal of Personality and Social Psychology*, *57*(6), 1069–1081. 10.1037/0022-3514.57.6.1069

[CR48] Salsman, J., Lai, J.-S., Hendrie, H., Butt, Z., Zill, N., Pilkonis, P. A., et al. (2014). Assessing psychological well-being: Self-report instruments for the NIH Toolbox. *Quality of Life Research,**23*(1), 205–215. 23771709 10.1007/s11136-013-0452-3 PMC3883907

[CR49] Schulz, R., & Heckhausen, J. (1996). A life span model of successful aging. *American Psychologist*, *51*(7), 702–714. 10.1037/0003-066x.51.7.702 8694390 10.1037//0003-066x.51.7.702

[CR50] Shanahan, L., Steinhoff, A., Bechtiger, L., Murray, A. L., Nivette, A., Hepp, U., Ribeaud, D., & Eisner, M. (2020). Emotional distress in young adults during the COVID-19 pandemic: Evidence of risk and resilience from a longitudinal cohort study. *Psychological Medicine*, *52*(5), 824–833. 10.1017/s003329172000241x 32571438 10.1017/S003329172000241X PMC7338432

[CR52] Swift, A. U., Bailis, D. S., Chipperfield, J. G., Ruthig, J. C., & Newall, N. E. (2008). Gender differences in the adaptive influence of folk beliefs: A longitudinal study of life satisfaction in aging. *Canadian Journal of Behavioural Science / Revue Canadienne Des Sciences Du Comportement,**40*(2), 104–112. 10.1037/0008-400x.40.2.104

[CR51] Swift, A. U., & Chipperfield, J. G. (2013). Secondary control belief combinations (adjustment and acceptance) and well-being in older adults. *Canadian Journal on Aging / La Revue Canadienne Du Vieillissement,**32*(4), 349–359. 10.1017/s0714980813000391 24063550 10.1017/S0714980813000391

[CR53] Thompson, S. C., Nanni, C., & Levine, A. (1994). Primary versus secondary and central versus consequence-related control in HIV-positive men. *Journal of Personality and Social Psychology,**67*(3), 540–547. 10.1037/0022-3514.67.3.540 7965603 10.1037//0022-3514.67.3.540

[CR54] Thompson, S. C., Thomas, C., Rickabaugh, C. A., Tantamjarik, P., Otsuki, T., Pan, D., Garcia, B. F., & Sinar, E. (1998). Primary and secondary control over age-related changes in physical appearance. *Journal of Personality,**66*(4), 583–605. 10.1111/1467-6494.00025 9728417 10.1111/1467-6494.00025

[CR55] Toyama, M. (2022). Longitudinal and age-related implications of primary and secondary control for hedonic and eudaimonic well-being. *Journal of Happiness Studies,**23*(5), 2313–2336. 10.1007/s10902-022-00501-w

[CR56] Vilar-Compte, M., Hernández-F, M., Gaitán-Rossi, P., Pérez, V., & Teruel, G. (2022). Associations of the COVID-19 pandemic with social well-being indicators in Mexico. *International Journal for Equity in Health*. 10.1186/s12939-022-01658-9 35597958 10.1186/s12939-022-01658-9 PMC9123783

[CR57] Wadsworth, M. E., Raviv, T., Compas, B. E., & Connor-Smith, J. K. (2005). Parent and adolescent responses to poverty related stress: Tests of mediated and moderated coping models. *Journal of Child and Family Studies,**14*(2), 283–298. 10.1007/s10826-005-5056-2

[CR58] Wang, M. T., Toro, J. D., Scanlon, C. L., Schall, J. D., Zhang, A. L., Belmont, A. M., Voltin, S. E., & Plevniak, K. A. (2021). The roles of stress, coping, and parental support in adolescent psychological well-being in the context of COVID-19: A daily-diary study. *Journal of Affective Disorders,**294*, 245–253. 10.1016/j.jad.2021.06.082 34303304 10.1016/j.jad.2021.06.082 PMC8433600

[CR59] Watson, D., Clark, L. A., & Tellegen, A. (1988). Development and validation of brief measures of positive and negative affect: The panas scales. *Journal of Personality and Social Psychology,**54*(6), 1063–1070. 10.1037/0022-3514.54.6.1063 3397865 10.1037//0022-3514.54.6.1063

[CR60] Whelan, C. T., Layte, R., Maitre, B., & Nolan, B. (2001). Income, deprivation, and economic strain. An analysis of the European Community Household Panel. *European Sociological Review,**17*(4), 357–372. 10.1093/esr/17.4.357

[CR62] Wrosch, C., Barlow, M. A., & Kunzmann, U. (2018). Age-related changes in older adults’ anger and sadness: The role of perceived control. Psychology and Aging, 33(2), 350. 10.1037/pag0000229 10.1037/pag0000229 29658752

