# Supplementary material for: Dynamic benefits of finding the silver lining: secondary control as a buffer against declines in well-being during COVID-19
Source: Motiv Emot. 2026 Feb 6;50(4):688–701. doi: 10.1007/s11031-026-10195-9 (PMC13042480; doi:10.1007/s11031-026-10195-9)
Supplement: Supplementary file 1 [file 11031_2026_10195_MOESM1_ESM.docx]

**Supplementary Material For:**

Dynamic Benefits of Finding the Silver Lining: Secondary Control as a Buffer Against Declines in Well-Being During COVID-19

**Supplemental Analysis: 3-Way Interaction**

Due to prior research that suggests differing salience and benefits of SC across the adult lifespan, a supplemental analysis examined a 3-way, cross-level interaction between within-person SC, within-person hardship, and between-person age on the well-being outcomes. The models were specified as described in the moderated effects section, with the addition of the interaction between SC, financial hardship, and baseline age (as well as all lower-order interactions between these variables). Results are summarized in Table S1. The 3-way interaction was small, but significant, in predicting negative and positive affect.

Simple slopes were used to probe the 3-way interaction and examine the within-person effects of SC on changes positive and negative affect, as moderated by within-person changes in financial hardship and between-person differences in age (+/- 1 SD). The resulting graphs for positive and negative affect can be seen in Figures S1 and S2, respectively.

The effect of SC on positive affect was probed for occasions when financial hardship increased vs. declined (+/-1 SD) for both younger and older adults. For younger adults, increases in SC strongly predicted corresponding increases in PA on occasions when individuals experienced *less hardship* than usual (*γ* = 0.27, *SE* = .086, *p* = .002). In contrast, increases in SC were not associated with changes in PA on occasions when younger adults experienced more hardship than usual (*γ* = 0.08, *SE* = .086, *p* = .381). This pattern was reversed for older adults, such that increases in SC strongly predicted corresponding increases in PA on occasions when individuals experienced *more hardship* than usual (*γ* = 0.35, *SE* = .083, *p* < .001). Increases in SC were not associated with changes in PA on occasions when older adults experienced less hardship than usual (*γ* = 0.12, *SE* = .093, *p* = .204).

A similar pattern was observed for NA. For younger adults, increases in SC predicted corresponding decreases in NA on occasions when individuals experienced less hardship than usual (*γ* = -0.19, SE = .077, *p* = .014). In contrast, increases in SC were not associated with changes in NA on occasions when younger adults experienced more hardship than usual (*γ* = -0.07, *SE* = .078, *p* = .343). This pattern was reversed for older adults, such that increases in SC strongly predicted corresponding decreases in NA on occasions when individuals experienced more hardship than usual (*γ* = -0.24, *SE* = .075, *p* = .001). Increases in SC were not associated with changes in NA on occasions when they experienced less hardship than usual (*γ* = 0.08, *SE* = .075, *p* = .317). This indicates that the impact of SC on hedonic well-being, at least, may be felt differently across the adult lifespan.

**Table 4:** Moderated Effects of Secondary Control on Well-Being as a Function of Financial Hardship and Age

| Variable | DP (SE) | LS (SE) | MN (SE) | NA (SE) | PA (SE) | PG (SE) | PS (SE) |
| --- | --- | --- | --- | --- | --- | --- | --- |
| **Level-1 (wp)** |  |  |  |  |  |  |  |
| Intercept | 1.98 (0.03)** | 3.83 (0.08)** | 3.63 (0.04)** | 2.16 (0.04)** | 2.74 (0.04)** | 5.53 (0.05)** | 2.61 (0.04)** |
| Time | -0.01 (0.00)** | 0.00 (0.00) | 0.00 (0.00)** | -0.01 (0.00)** | 0.01 (0.00)** | -0.00 (0.00) | -0.00 (0.00)** |
| SC | -0.10 (0.03)** | 0.14 (0.05)* | 0.19 (0.04)** | -0.11 (0.04)* | 0.20 (0.04) ** | 0.23 (0.05)** | -0.13 (0.03)** |
| Hardship | 0.06 (0.03)* | -0.13 (0.05)* | -0.02 (0.04) | 0.09 (0.04)* | -0.11 (0.04)* | 0.03 (0.05) | 0.11 (0.03)** |
| SC x Hardship | -0.08 (0.07) | 0.08 (0.14) | -0.16 (0.10) | -0.13 (0.11) | 0.01 (0.12) | 0.31 (0.14)* | -0.22 (0.09)* |
| **Level-2 (bp)** |  |  |  |  |  |  |  |
| Age | -0.01 (0.00)** | -0.00 (0.00) | -0.00 (0.00) | -0.01 (0.00)** | 0.00 (0.00) | -0.01 (0.00)** | -0.01 (0.00)** |
| Sex | 0.24 (0.07)** | -0.23 (0.16) | -0.07 (0.09) | 0.36 (0.09)** | -0.30 (0.08)** | 0.20 (0.10)* | 0.33 (0.08)** |
| Edu | -0.02 (0.01) | 0.12 (0.03)** | 0.03 (0.02) | -0.02 (0.02) | 0.03 (0.02) | 0.03 (0.02) | -0.02 (0.02) |
| Inc | 0.00 (0.01) | 0.04 (0.02) | 0.01 (0.01) | -0.00 (0.01) | -0.00 (0.01) | 0.02 (0.02) | 0.00 (0.01) |
| SC(bp) | -0.32 (0.04)** | 0.89 (0.10)** | 0.61 (0.05)** | -0.35 (0.05)** | 0.44 (0.05)** | 0.62 (0.06)** | -0.36 (0.05)** |
| Hardship(bp) | 0.33 (0.06)** | -0.91 (0.15)** | -0.24 (0.08)** | 0.42 (0.08)** | -0.32 (0.08)** | -0.15 (0.10) | 0.47 (0.07)** |
| SC x Age | 0.00 (0.00) | 0.00 (0.00) | 0.00 (0.00) | 0.00 (0.00) | 0.00 (0.00) | 0.01 (0.00)* | -0.00 (0.00) |
| Hardship x Age | -0.00 (0.00) | 0.00 (0.00) | -0.00 (0.00) | -0.00 (0.00) | 0.00 (0.00) | 0.00 (0.00) | 0.00 (0.00) |
| SC x Hardship x Age | **-0.01 (0.00)** | **0.01 (0.01)** | **0.01 (0.01)** | **-0.02 (0.01)*** | **0.02 (0.01)*** | **0.01 (0.01)** | **-0.01 (0.01)** |

*Note*. wp = within-person. bp = between-person. DP = depressive symptoms, LS = life satisfaction, NA = negative affect, PA = positive affect, PG = personal growth, and PS = perceived stress. wp = within-person and bp = between-person. Within-person variables were person-mean centered, between-person variables were grand-mean centered. The interaction between within-person secondary control, within-person hardship, and age was found to be significant for positive and negative affect. Parameter estimates for the interaction of primary interest (baseline age x within-person SC x within-person hardship) are shown in bold font.

*p<0.05, **p<0.005

**Figure S1:** Impact of Secondary Control on Positive Affect, Moderated by Financial Hardship and Age.

*Note*. This graph depicts the within-person relationship between changes in secondary control and positive affect for younger vs. older adults on occasions when the participant is experiencing less financial hardship (darker, dotted) or greater financial hardship (lighter, straight). Ribbons indicate 95% CI. This interaction is separated by younger (age -1 SD) and older (age +1 SD) participants.

**Figure S2:** Impact of Secondary Control on Negative Affect, Moderated by Financial Hardship and Age.

*Note*. This graph depicts the within-person relationship between changes in secondary control and negative affect for younger vs. older adults on occasions when the participant is experiencing less financial hardship (darker, dotted) or greater financial hardship (lighter, straight). Ribbons indicate 95% CI. This interaction is separated by younger (age -1 SD) and older (age +1 SD) participants.
